# DiMANI: diffusion MRI for anatomical nuclei imaging—Application for the direct visualization of thalamic subnuclei

**DOI:** 10.3389/fnhum.2024.1324710

**Published:** 2024-02-19

**Authors:** Rémi Patriat, Tara Palnitkar, Jayashree Chandrasekaran, Karianne Sretavan, Henry Braun, Essa Yacoub, Robert A. McGovern, Joshua Aman, Scott E. Cooper, Jerrold L. Vitek, Noam Harel

**Affiliations:** ^1^Center for Magnetic Resonance Research, Department of Radiology, University of Minnesota, Minneapolis, MN, United States; ^2^Graduate Program in Neuroscience, University of Minnesota, Minneapolis, MN, United States; ^3^Department of Neurosurgery, University of Minnesota, Minneapolis, MN, United States; ^4^Department of Neurology, University of Minnesota, Minneapolis, MN, United States

**Keywords:** thalamus, thalamic subnuclei, thalamus parcellation, diffusion MRI, direct visualization, DiMANI, DBS

## Abstract

The thalamus is a centrally located and heterogeneous brain structure that plays a critical role in various sensory, motor, and cognitive processes. However, visualizing the individual subnuclei of the thalamus using conventional MRI techniques is challenging. This difficulty has posed obstacles in targeting specific subnuclei for clinical interventions such as deep brain stimulation (DBS). In this paper, we present DiMANI, a novel method for directly visualizing the thalamic subnuclei using diffusion MRI (dMRI). The DiMANI contrast is computed by averaging, voxelwise, diffusion-weighted volumes enabling the direct distinction of thalamic subnuclei in individuals. We evaluated the reproducibility of DiMANI through multiple approaches. First, we utilized a unique dataset comprising 8 scans of a single participant collected over a 3-year period. Secondly, we quantitatively assessed manual segmentations of thalamic subnuclei for both intra-rater and inter-rater reliability. Thirdly, we qualitatively correlated DiMANI imaging data from several patients with Essential Tremor with the localization of implanted DBS electrodes and clinical observations. Lastly, we demonstrated that DiMANI can provide similar features at 3T and 7T MRI, using varying numbers of diffusion directions. Our results establish that DiMANI is a reproducible and clinically relevant method to directly visualize thalamic subnuclei. This has significant implications for the development of new DBS targets and the optimization of DBS therapy.

## 1 Introduction

The thalamus is a centrally located brain structure involved in a myriad of sensory, motor, and cognitive processes with direct and indirect connections throughout the cortical brain. As such, it has been the focus of many neuroimaging studies with applications in movement, psychiatric, and other mental disorders [see Boelens Keun et al. (2021) for a review]. What gives the thalamus such diverse capabilities and roles is its heterogeneous constitution comprising many smaller subnuclei, each with its own set of structural connections. While historically thalamus subdivision and nomenclature have differed across many key histological and postmortem reports (Mai and Majtanik, 2018), seven morphological groups have been identified. The anterior nuclear group is associated with visual memory and emotional, cognitive, and executive functions. The lateral group is involved in spatial navigation, limbic functions, and visual information processing. The ventral group partakes in motor functions, language, and somatosensory information processing. The intralaminar group is involved in attention regulation, salience, and arousal. The medial group is associated with vigilance, awareness, executive functioning, emotion processing, and memory. The reticular nucleus is involved in the generation of sleep spindles and other processes as theorized in the “searchlight hypothesis” (Crick, 1984). Finally, the posterior nuclear group processes and integrates sensory information from visual, auditory, and multisensory modalities (Boelens Keun et al., 2021).

Clinical treatments and therapies, such as deep brain stimulation (DBS) and focused ultrasound, take advantage of the different functions subserved by these different groups by targeting specific subsubnuclei which have functions related to a given disorder (see Supplementary Table 1). For example, the ventral intermediate nucleus of the thalamus (Vim), part of the ventral group, is a target for DBS and thalamotomy for tremor-related movement disorders such as essential tremor (ET) (Dallapiazza et al., 2019). The anterior thalamus has been extensively targeted for the treatment of refractory epilepsy (Bouwens van der Vlis et al., 2019), although it is not always clear whether it is the anteroventral (AV), the anterodorsal (AD) or even part of the ventralanterior (VA) nucleus that is being stimulated. The mediodorsal nucleus (MD), part of the medial group, has been tested as a target for obsessive-compulsive disorder (Maarouf et al., 2016). The centromedian (CM), part of the intralaminar group, is one DBS target for the treatment of Tourette's syndrome (Casagrande et al., 2019) and generalized epilepsy, a.k.a. Lennox-Gastaut syndrome (Aungaroon, 2022). Of note, the intralaminar subnuclei of the thalamus have also been targeted using DBS (Schiff et al., 2007) and low-intensity focused ultrasound (Monti et al., 2016) to restore routine behavior and consciousness in patients in a minimally conscious state following brain injury. Using DBS in the ventral posterior nucleus, part of the ventral group, has shown promise in the treatment of neuropathic pain (Boccard et al., 2013). Many of these targets are also under consideration as targets for lesioning procedures such as thalamotomy and focused ultrasound. While many of these thalamic DBS approaches have demonstrated significant benefits to patients, others were unsuccessful. Further, even the standard DBS and lesioning approaches often suffer from variable patient outcomes. One reason for heterogenous results in clinical efficacy might stem from the difficulty to directly visualize and target specific subnuclei of the thalamus on clinical brain images as well as interindividual variability in the size, shape and geometric configuration of thalamic subnuclei.

The most commonly used magnetic resonance imaging (MRI) contrasts in the clinical setting (i.e. T1 and T2) do not show individual thalamic subnuclei due to poor contrast within the thalamus. Therefore, direct targeting for DBS using current clinical imaging protocols is extremely difficult. This has led surgical teams to use templates and atlases, which do not account for inter-individual anatomic variability, and are oftentimes derived from individuals outside of the patient's population demographics (e.g., age, disease state). Several groups have worked to develop new imaging methods for direct visualization, including susceptibility weighted imaging (SWI) (Abosch et al., 2010; Najdenovska et al., 2019), quantitative magnetic susceptibility mapping (QSM) (Deistung et al., 2013; Chiang et al., 2018), or white-matter nulled T1 imaging, such as FGATIR (Sudhyadhom et al., 2009; Hoch and Shepherd, 2022), 3D-EDGE (Middlebrooks et al., 2021), and WMn-MPRAGE (Su et al., 2019). However, these methods have drawbacks. Despite using a 7 Tesla (7T) MRI scanner, Najdenovska et al. (2019) demonstrated that direct visualization of the Vim in SWI images was not always possible, and QSM typically involves long acquisition times as well as complex imaging protocols and reconstruction algorithms making them difficult to use routinely in the clinic. The standard FGATIR acquisition—without expert parameter adjustments—can suffer from poor contrast at 7T due to B1 inhomogeneity (Tao et al., 2022). Additionally, 3D MRI acquisitions schemes, such as SWI and MPRAGE-based sequences, are extremely sensitive to motion, a substantial issue when working with movement disorder patients. Finally, several of these methods are not common practice in the clinical world as they require MR expertise and computational capabilities not available in most non-academic centers.

Diffusion MRI (dMRI) has been extensively used to attempt to uncover the sub-territories of the thalamus. The two main strategies involve tractography and clustering based on local fiber orientation. The former computes the connectivity of each voxel in the thalamus to multiple cortical regions of interest (ROIs) and assigns each voxel a label based on the relative connectivity strength to these ROIs (Behrens et al., 2003). The latter models local fiber orientation distributions or the dominant diffusion orientation at each voxel within the thalamus and then generates parcels via a clustering approach (Mang et al., 2012; Battistella et al., 2017; Iglehart et al., 2020). Tractography-based parcellation results, the most used dMRI method to date (Su et al., 2019; Iglehart et al., 2020), are heavily dependent on the targets being included in the tractography analysis. Inclusion or exclusion of one cortical region will change the results (e.g., final number of clusters, cluster assignment, border location). Additionally, these methods typically use a winner-take-all approach in which a voxel is assigned to a territory based on its relatively high connectivity to a target (e.g. motor cortex) (Behrens et al., 2003) regardless of the fact that it may also be highly connected to another region. In fact, many thalamic subnuclei are connected to multiple brain regions [Table 1 from Boelens Keun et al. (2021)].

Here we present a novel method to directly visualize the subnuclei of the thalamus that can be implemented on previously acquired dMRI datasets. The proposed method, termed DiMANI, is based on the voxelwise average of diffusion weighted volumes to enhance the anatomical contrast within the tissue, allowing for the direct distinction of thalamic subnuclei in individuals.

The reproducibility of the proposed method was evaluated in several ways. First, on the acquisition side, a unique dataset consisting of 8 scans of a single participant collected over a 3-year period were segmented and compared (test-retest). Second, on the image post-processing side, manual segmentations of thalamic subnuclei were quantitatively evaluated for both intra-rater as well as inter-rater reliability. Third, on the clinical side, DiMANI was evaluated in data from several DBS patients and qualitatively correlated with electrode localization and clinical observations. Finally, we show that DiMANI provides similar contrast at 3T and 7T with varying number of diffusion directions.

## 2 Methods

### 2.1 Subjects

Six ET patients were enrolled from the University of Minnesota DBS program. Inclusion criteria required patients to have a diagnosis of Essential Tremor and be suitable candidates for Vim-DBS surgery. The study did not interfere or change the patients' routine treatment protocol except for adding one extra 7T MRI scan prior to surgery. The patient cohort consisted of four males and two females with an average age of 61.2 ± 12.3 years and an average disease duration of 22.5 ± 10.9 years. One recruit from the University of Minnesota volunteer pool served as a healthy control and was scanned eight times (male, 53 years at the time of the first scan and 56 years at the eighth). Finally, one healthy control, subject 100610 (male, 26–30 years), and one patient, subject 85236 (male, 69 years), were downloaded from the Human Connectome Project (HCP) and from the Parkinson's Progression Markers Initiative (PPMI) databases, respectively (https://www.humanconnectome.org, https://www.ppmi-info.org). The study was approved by the Institutional Review Board at the University of Minnesota and informed consent was obtained from all participants prior to inclusion in the study. All experiments were performed in accordance with relevant guidelines and regulations.

### 2.2 MRI acquisition and processing

All University of Minnesota participants were scanned at the Center for Magnetic Resonance Research on a 7T-Terra MRI scanner using SC72 gradients capable of 70 mT/m and a 200 T/m/s slew rate, driven by a Siemens console (Erlangen, Germany). The images were acquired with a 32-element head array coil (Nova Medical, Inc, Burlington, MA). Diffusion-weighted images covering the whole brain were acquired using 50 directions, b-value = 1,500 s/mm^2^, 4 additional b0-volumes, 1.25 mm isotropic voxels, multi-band (MB) = 2, parallel acceleration (GRAPPA) = 2. The diffusion images were acquired twice, each with different phase encoding directions: anterior-posterior and posterior-anterior for a total of 13 minutes. Additionally, for our control participant, an additional session included an FGATIR (whole-brain, 0.8 mm isotropic, TR/TE = 3,000/2ms, TI = 430ms, GRAPPA = 2, 8 min 43 s), SWI (0.4x0.4x08 mm interpolated to 0.2x0.2x0.8 mm, TR/TE = 210/14ms, GRAPPA = 2, 7 min 33 s), and a multi-echo MP2RAGE in order to generate a synthetic WMn-MPRAGE image to obtain the THOMAS atlas output (Su et al., 2019) (0.66 mm isotropic, TR = 6,000 ms, TE = 1.8/3.6/5.5/7.4 ms, TI = 750/2950 ms, GRAPPA = 3, 12 min 20 s).

dMRI preprocessing steps included: motion, susceptibility, and eddy current distortions correction using FSL's eddy and topup algorithms (Andersson et al., 2003). The DiMANI images were generated by computing the mean, voxelwise, of the diffusion weighted volumes (e.g., only b > 100 volumes were kept). For visualization purposes, DiMANI images were equalized using an adaptive histogram equalization (https://github.com/VincentStimper/mclahe).

The HCP datasets were acquired at 3T and 7T. The protocols have been published elsewhere (Sotiropoulos et al., 2013, 2016; Moeller et al., 2021). The 3T HCP diffusion MRI dataset was acquired at 1.25 mm isotropic resolution with 3 shells (*b* = 1,000, 2,000, 3,000 s/mm^2^) and a total of 288 diffusion volumes (18 b-0 volumes). The 7T HCP diffusion dataset was acquired at 1 mm isotropic resolution with 2 shells (*b* = 1,000 and 2,000 s/mm^2^) and a total of 143 directions (15 b-0 volumes). The PPMI dataset was acquired using a 3T MRI scanner with 2 mm isotropic voxels, *b* = 1,000 s/mm^2^ and 64 directions plus one b-0 volume. For the HCP and PPMI datasets, the downloaded datasets were already processed.

### 2.3 Thalamus segmentation

Manual segmentation of the thalamus and its subnuclei was carried out using the contrast information from the average diffusion image of each participant separately. There is a wide variety of thalamic subnuclei nomenclature and subdivisions in the literature arising from differences in the methodology used to visualize the thalamus. Therefore, we could not conduct segmentation of the subnuclei following all available atlases. The THOMAS atlas is arguably the current gold standard for thalamic subnuclei subdivision using MRI (Su et al., 2019). One advantage of using this atlas is its distribution with code that enables automatic parcellation of the thalamus based on the subject's images. This facilitates comparison of our manual segmentations to a state-of-the-art tool. Its nomenclature and subdivision are largely based on that of the Morel atlas (Morel et al., 1997). The Morel atlas contains many more subnuclei than the THOMAS because it is based on histology, rather than MRI which has much lower resolution. In this study, we will show results based on both atlases and nomenclatures. The THOMAS atlas subnuclei segmentations come from the output of the inference code (https://github.com/thalamicseg/thomas_new) based on a participant synthetic WMn-MPRAGE computed from the multi-echo MP2RAGE image (Su et al., 2019). The Morel atlas was non-linearly warped to the subject's native space using the HCP pipelines (FSL FLIRT and FNIRT) (Glasser et al., 2013).

### 2.4 Reproducibility of the visualization

One healthy control was scanned 8 times over the course of 26 months on two different 7T MRI scanners at the University of Minnesota (Siemens MAGNETOM actively shielded and Siemens MAGNETOM 7T Terra) employing similar acquisition protocols as described above. Manual segmentation was performed on each dataset separately. The resulting segmentations were registered to one of the eight datasets using affine registrations and Dice coefficients (DCs) were computed for each nucleus inside of Slicer3D (Fedorov et al., 2012). Additionally, manual segmentation was performed across three experienced raters independently on five ET patients and an inter-rater DC was also computed.

### 2.5 Clinical validation

The purpose of this experiment was to verify the accuracy of our manual segmentation of the Vim and its surrounding subnuclei by comparing them with the placement of the DBS electrodes. Additionally, using the surgical notes and the reconstruction of the microelectrode recordings (MER) trajectories, we aimed to verify the segmentation of the ventral posterior lateral (VPL) region [also called Vc (Ilinsky and Kultas-Ilinsky, 2002)]. A typical targeting methodology at our center is to use a “cross-shaped” BenGun affixed to the head frame and aligned in the patient's anterior-posterior direction. MER is then performed in the posterior and center positions and cell firing patterns and receptive fields are identified. VPL cells are recognizable by tactile receptive fields. Distance from the posterior position for lead implantation depends on where Vc is identified. Roughly 4 weeks post-surgery, a postop computed tomography (CT) image is acquired, and the DBS system is turned on and optimized for patient benefits against side effects. The CT is then non-linearly registered to the MR images using Elastix (Klein et al., 2010) in order to locate the exact location of the DBS electrode (s) (shaft and contacts) within the patient's own anatomy. Additionally, we extract the depths at which VPL cells were identified intra-operatively and map them in 3D with respect to the final lead location using 3D Slicer (Fedorov et al., 2012).

### 2.6 DTI and tractography validation

The purpose of this qualitative experiment is to verify the accuracy of our manual segmentations against probabilistic and deterministic tractography results. Following dyads computation using bedpostx, probtrackx2 (Behrens et al., 2003) (with the –os2t option) was used to generate probabilistic tractography maps with each thalamus as a seed and cortical regions as targets. The cortical targets were: M1, S1, premotor, supplemental motor area (SMA), limbic, associative, parietal, occipital, and temporal. This resulted in one thalamus map per ipsilateral cortical target (9 per side). Each map was thresholded to its 99th percentile to find the thalamic location of highest connectivity to each cortical region. This location was overlaid on the manual segmentation. For the deterministic experiment, the preprocessed data were imported into DSI studio (http://dsi-studio.labsolver.org) along with the manual segmentations. A deterministic fiber tracking algorithm (Yeh et al., 2013) was used with augmented tracking strategies (Yeh, 2020) to improve reproducibility. Each manually segmented thalamic nucleus was used as a seed with the other regions set as regions of avoidance, with the exception of the intralaminar subnuclei for which no regions of avoidance were set. Additionally, a mid-sagittal plane was created from the bottom of the thalamus to the top of the cortex and used as a region of avoidance to minimize cross-hemispheric streamlines. Tracking was stopped once 5,000 streamlines were created. Tracks with lengths shorter than 50 or longer than 150 mm were discarded. Other parameters were set to default.

## 3 Results

### 3.1 Nucleus visualization

**Figure 1:**
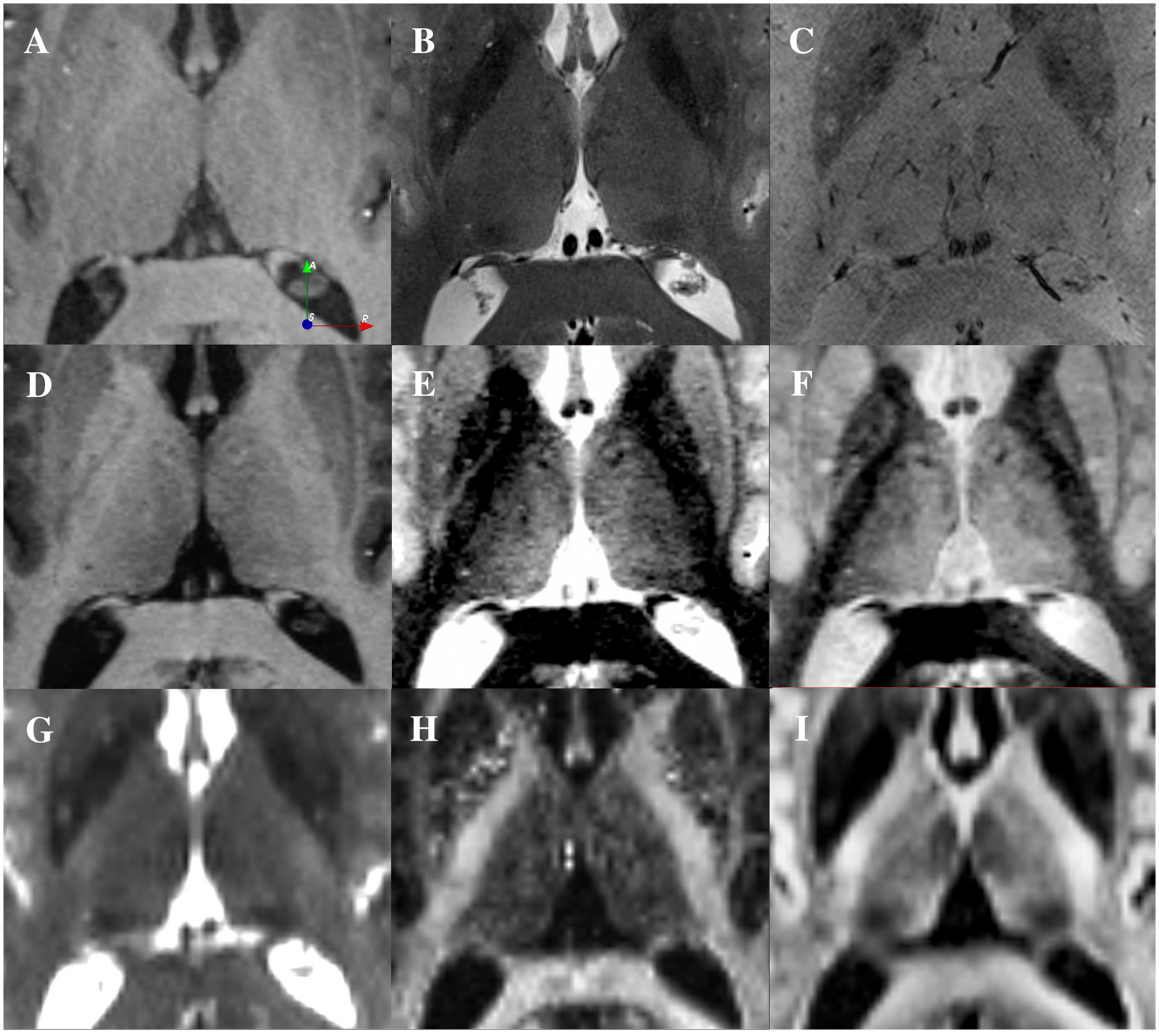
Visualization of the thalamus using different contrasts at 7T from a single participant. **(A)** T1-weighted image (0.6 mm isotropic). **(B)** T2-weighted image (0.4x0.4x1 mm). **(C)** SWI image (0.4x0.4x0.8 mm). **(D)** Multi-echo MP2RAGE (0.7 mm isotropic). **(E)** Synthetic WMn-MPRAGE reconstructed from the multi-echo MP2RAGE (0.7 mm isotropic). **(F)** FGATIR image (0.8 mm isotropic). **(G)** Average across 4 B0 images from a dMRI dataset (1.25 mm isotropic). **(H)** Fractional Anisotropy computed the dMRI data (1.25 mm isotropic). **(I)** DiMANI image reconstructed from the dMRI dataset (1.25 mm isotropic).

Panels of Figure 1 display one axial slice, from a single participant, through the thalamus for MRI contrasts commonly used to visualize the thalamus. On the T1 and T2 weighted and fractional anisotropy (FA) images (obtained with dtifit), little to no detail is visible within the structure (Figures 1A, B, H, respectively). The B0 image (Figure 1G) shows more information, especially for the MD and posteriormost regions of the thalamus (pulvinar and VPL). The FGATIR and WMn-MPRAGE images show details in the lateral and anterior portions of the thalamus (Figures 1F, E). However, while the center of mass of some subnuclei can be inferred, most of these images do not enable reliable visualization of the borders of most subnuclei as described in atlases such as THOMAS and Morel.

**Figure 2:**
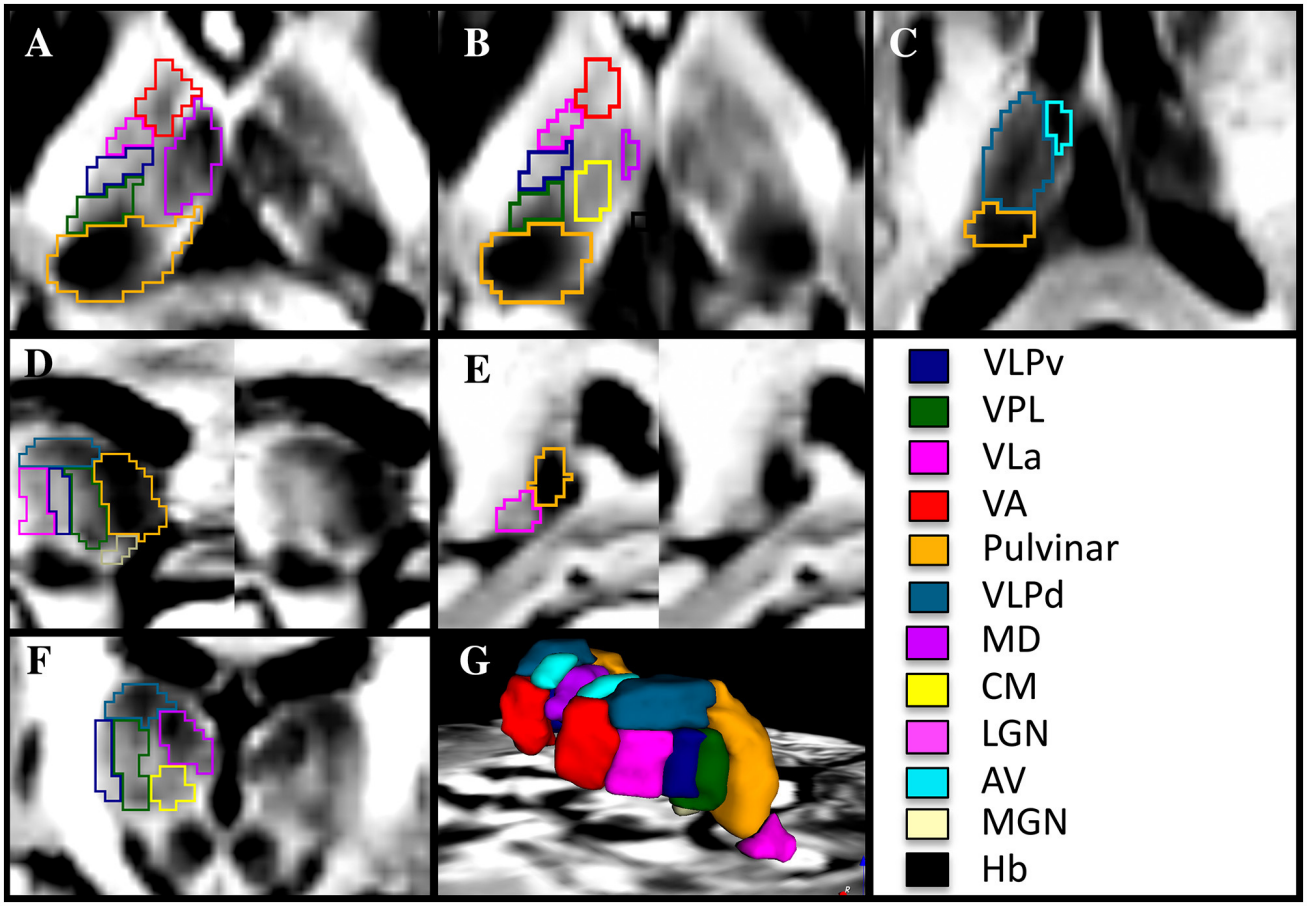
Manual segmentation of thalamic subnuclei using a 7T DiMANI image following conventions from the THOMAS atlas in axial **(A–C)**, sagittal **(D, E)**, coronal **(F)**, and in 3D **(G)** in a healthy volunteer. VLPv, ventral portion of the ventral lateral posterior nucleus; VLPd, dorsal portion of the ventral lateral posterior nucleus; VPL, Ventral posterior lateral nucleus; VLa, Ventral lateral anterior nucleus; VA, Ventral anterior nucleus; MD, Mediodorsal nucleus; CM, Centromedian nucleus; LGN, Lateral geniculate nucleus; AV, Anterior ventral nucleus; MGN, Medial geniculate nucleus; Hb, Habenular nucleus.

In contrast to the common MRI protocols that exhibit relatively flat or minimal details (Figure 1), the DiMANI method provides enhanced dynamic range and enables direct visualization of thalamic subnuclei (Figure 1I), including their borders, as seen in Figures 2A–F.

**Figure 3:**
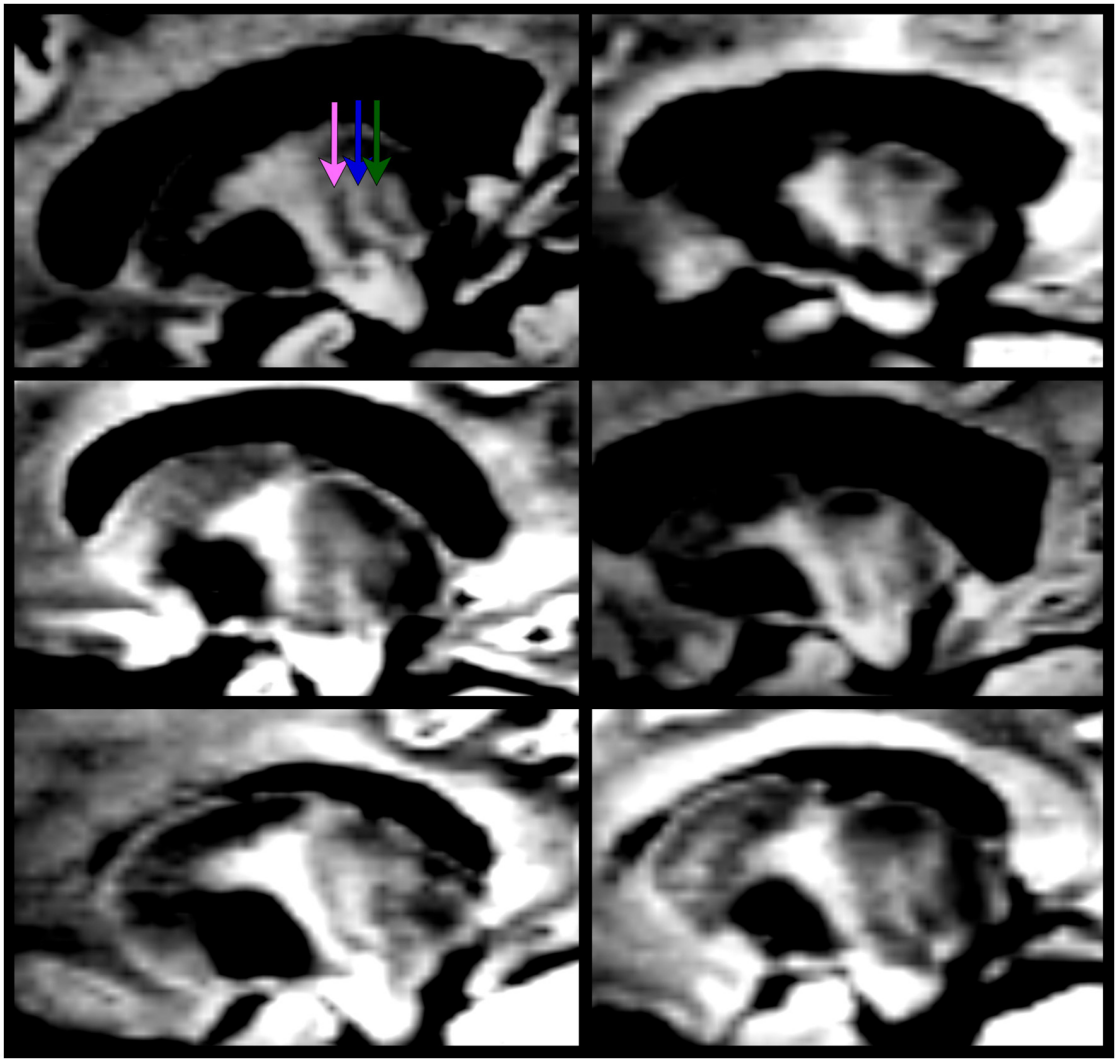
The “zebra pattern” is present in all ET patients enrolled in this study (using the 7T DiMANI image). The pink arrow represents the location of VLa; the blue arrow, VLPv; the green arrow, VPL.

The optimal view for manually segmenting subnuclei depends on the specific subnuclei being segmented. For example, the sagittal orientation proved key in segmenting the VLa, VLPv, and VPL as stripes of alternating dark and bright signals forming a “zebra pattern” were observed. This “zebra pattern” was visible on all our participants, including across ET patients (Figure 3).

The VLPd appears as a dark structure separated from the VLPv, VLa, and VPL in the coronal and sagittal views. The MD, appearing very dark, is visible and separated in all orientations from its bright to light gray surroundings, which are comprised of the internal medullary lamina laterally, the stria medullaris medially and ventral regions including the CM (ventral lateral). The separation between MD and CM is most visible in the coronal view. The pulvinar, darker than the VPL located at its anterior border, is best segmented in the axial view and corrected in the sagittal view. The anteriormost part of the VA is segmented in the coronal view while its borders with the lighter internal medullary lamina and dark VLa are segmented in the axial view. The very dark AV, situated at the most dorsal anterior and medial portion of the thalamus, is segmented in the axial and coronal views. The Hb is very small and is better visualized in the axial and coronal views. The lateral and medial geniculate subnuclei are both lighter than their shared neighbor, the pulvinar. It should be noted here that the MTT was not visible in all slices, hence manual segmentation of this white matter structure was not performed. This is likely due to image resolution (1.25 mm isotropic) with respect to the shape and volume of the structure.

**Figure 4:**
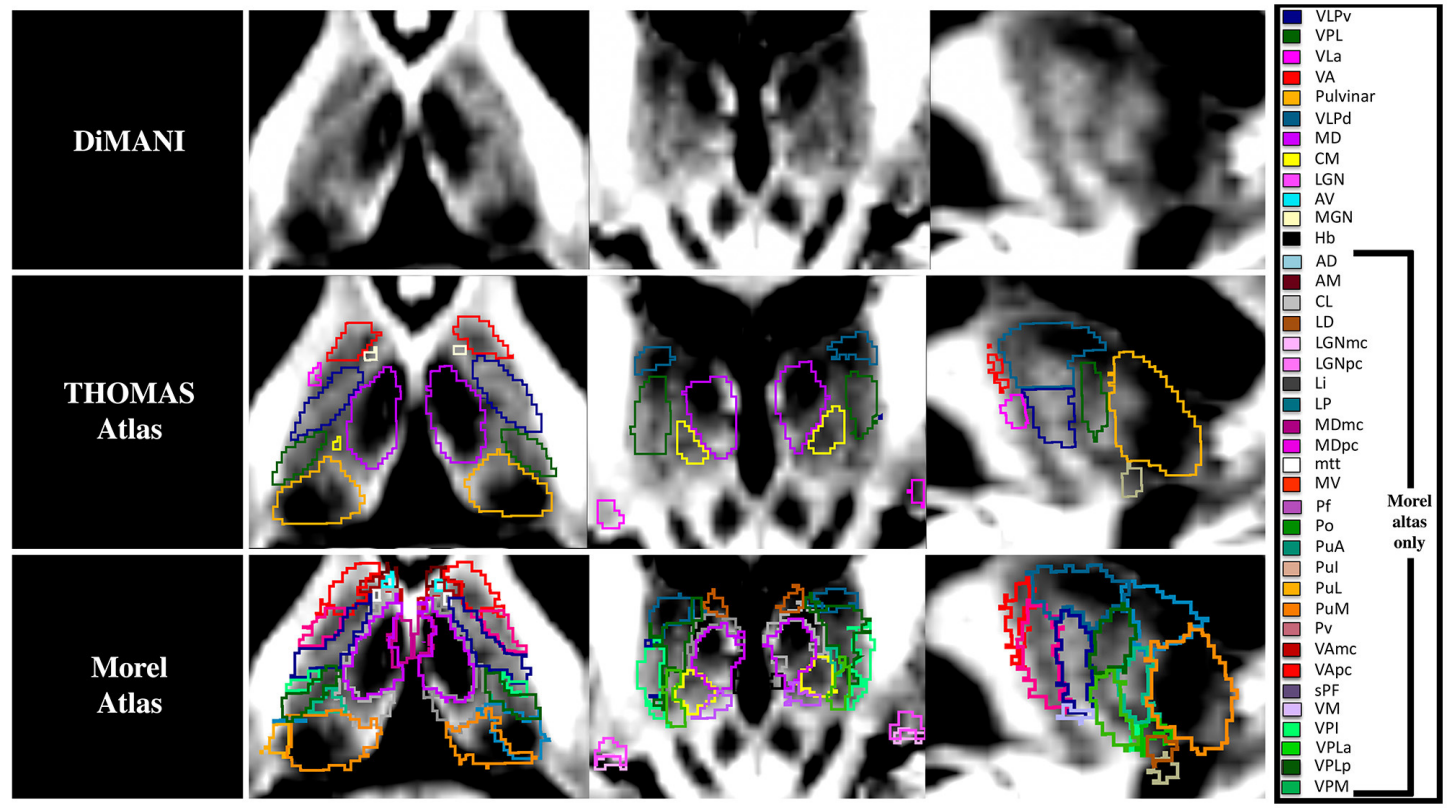
The DiMANI contrast obtained at 7T shows information that follows known anatomical organization from common atlases. All abbreviations are based on the Morel atlas (Morel et al., 1997).

Figure 4 shows that the DiMANI contrast corresponds to the overall organization of the THOMAS and Morel atlases (results are overlayed on the DiMANI image). Of note, the DiMANI contrast also enables visualization of subnuclei present in the Morel atlas but not in the THOMAS atlas. Of the many additional subnuclei, we were able to identify structures that likely correspond to the CL, PuA, LP, VPM, VPI, MDmc, MDpc, LGNmc, LGNpc, Pf, and sPF subnuclei.

### 3.2 Reproducibility

**Figure 5:**
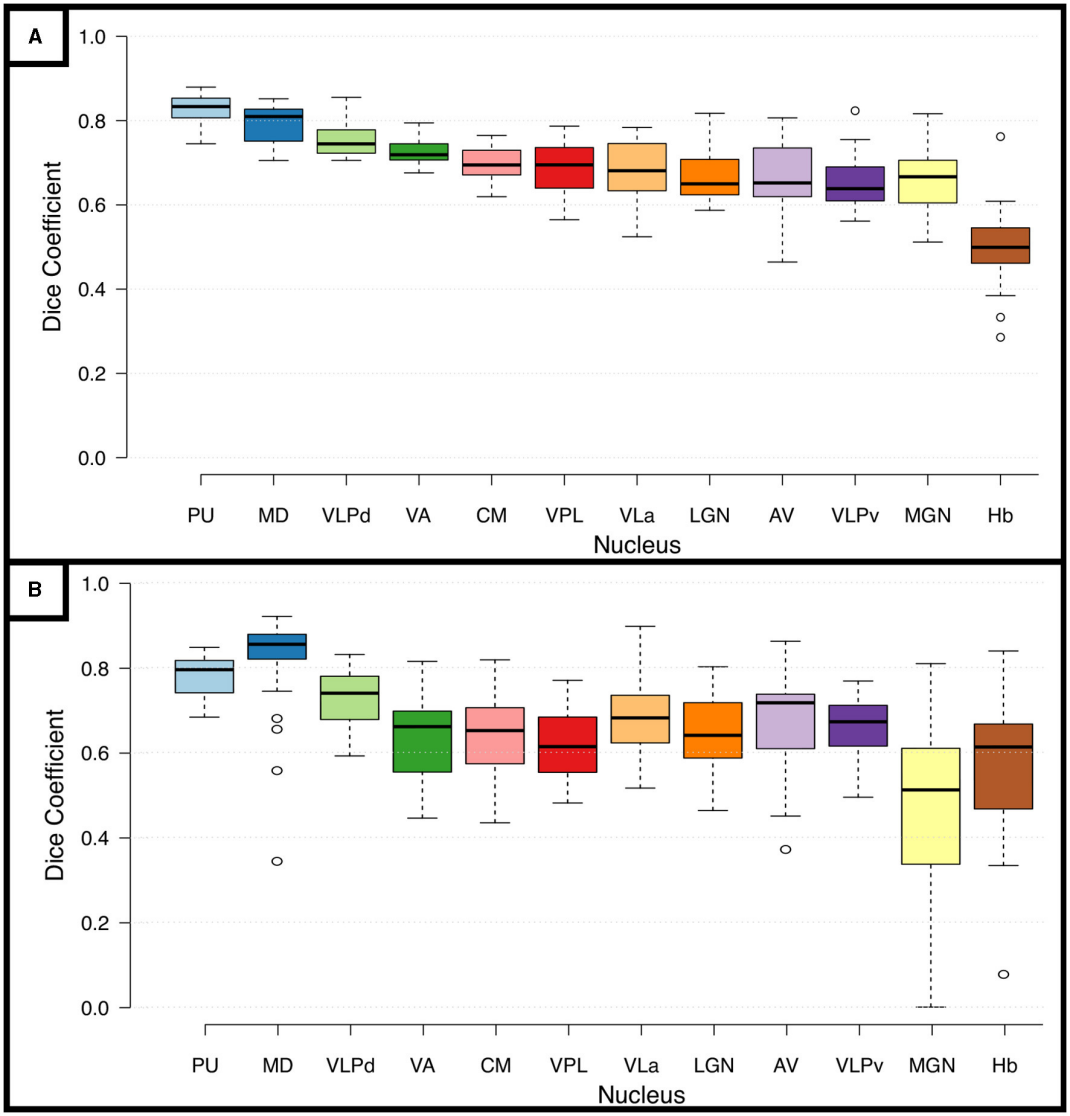
Reproducibility and Reliability of the manual segmentations using the DiMANI contrast. **(A)** Shows the dice coefficients from the manual segmentations performed on eight scans from the same participant. **(B)** Shows the inter-rater dice coefficients computed from manual segmentations of 5 patients from three raters (totaling 30 DC per subnuclei: 5patients x 2 hemispheres x 3 rater combinations).

To test the reliability of the segmentations based on the DiMANI contrast we calculated the overlap between manual segmentations performed on 8 scans of the same individual. Figure 5A shows the Dice coefficients (DCs) for the manual segmentation of the twelve visible subnuclei following the THOMAS atlas as a guide. This resulted in fourteen DCs per subnuclei (seven left and right). The averaged DCs were all between 0.62 and 0.85 except for the small Hb nucleus (DC = 0.50). Figure 5B shows the results for the inter-rater reliability. Each of the twelve subnuclei were segmented independently by three raters yielding in 120 manual segmentations per rater (12[subnuclei] x 2[sides] x 5[patients]). This resulted in comparable DCs to those obtained from the test-retest analysis (average DCs ranging from 0.46 to 0.81).

### 3.3 Clinical and imaging validation

**Figure 6:**
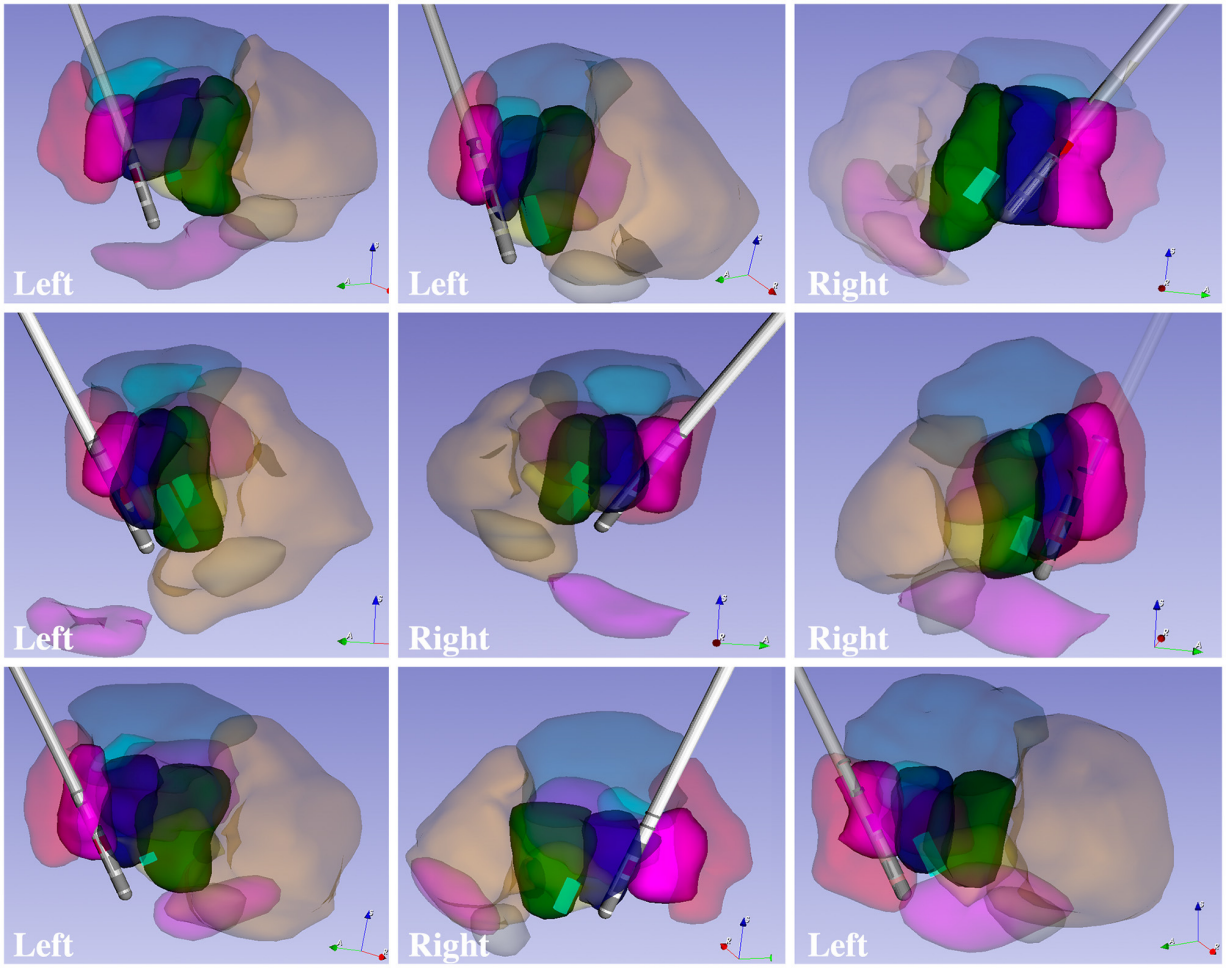
3D models of patient-specific thalamic segmentations combined with DBS lead and VPL cell locations for six patients totaling nine leads.

The goal of any new method is to provide added value to clinical and research approaches. Here, we show that the DiMANI contrast allows the segmentations and creation of 3D patient-specific models that depict the location of thalamic subnuclei. We leveraged neurophysiological data to test the validity of the DiMANI-based models by correlating the locations of the DBS electrodes and the stimulating contacts with the target subnuclei. Figure 6 shows that all nine active DBS contacts were at or near the VLPv-VLa border, which is consistent with expected lead locations based on our center's targeting approach. Additionally, 11 of 12 MER tracks displaying activity interpreted as VPL cells overlapped fully with the manual segmentations of the VPL. The twelfth was found just anterior to VPL within VLPv.

**Figure 7:**
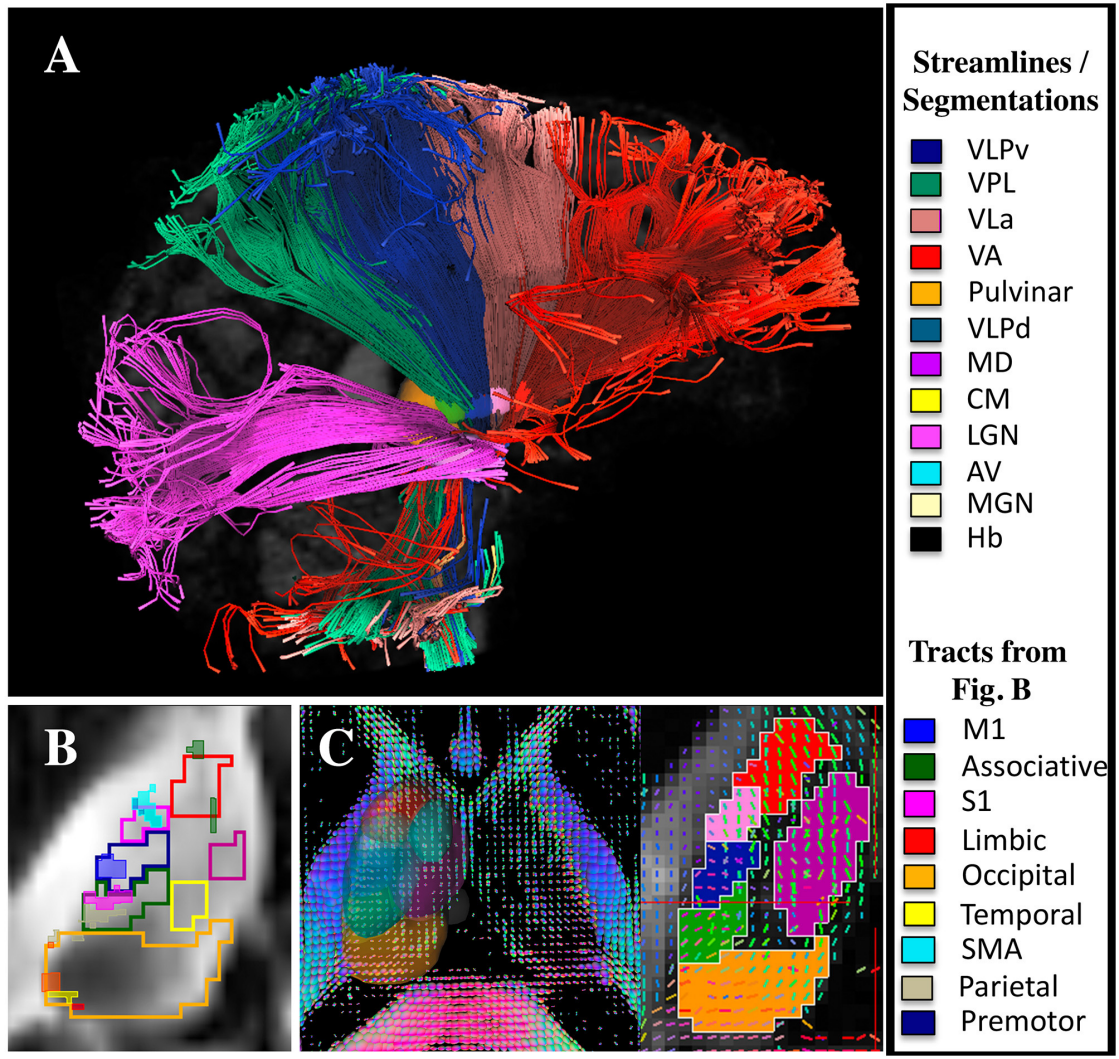
DTI and tractography validation. **(A)** Deterministic tractography results from the LGN, VPL, VLPv, VLa, and VA regions (note that the tractography was performed without cortical parcellations). **(B)** Probabilistic tractography results showing the connectivity hotspots (thresholded at 99% of the distribution) between cortical regions and the thalamus overlaid on manual segmentations performed using the DiMANI contrast (note that the tractography was performed without the manual segmentations). **(C)** Manual segmentations overlaid on local fiber orientation computed with DSI studio.

Figure 7 shows that both probabilistic and deterministic tractography result in known connections between the manual segmentations and cortical regions. For example, the VLPv connects to M1, the VPL to S1, the LGN to visual areas, and the VLa to premotor and SMA regions. Additionally, some of the thalamic regions show different fiber orientations compared to their neighbors, as expected from local fiber orientation parcellation methods (Figure 7C).

### 3.4 Impact of acquisition parameters

**Figure 8:**
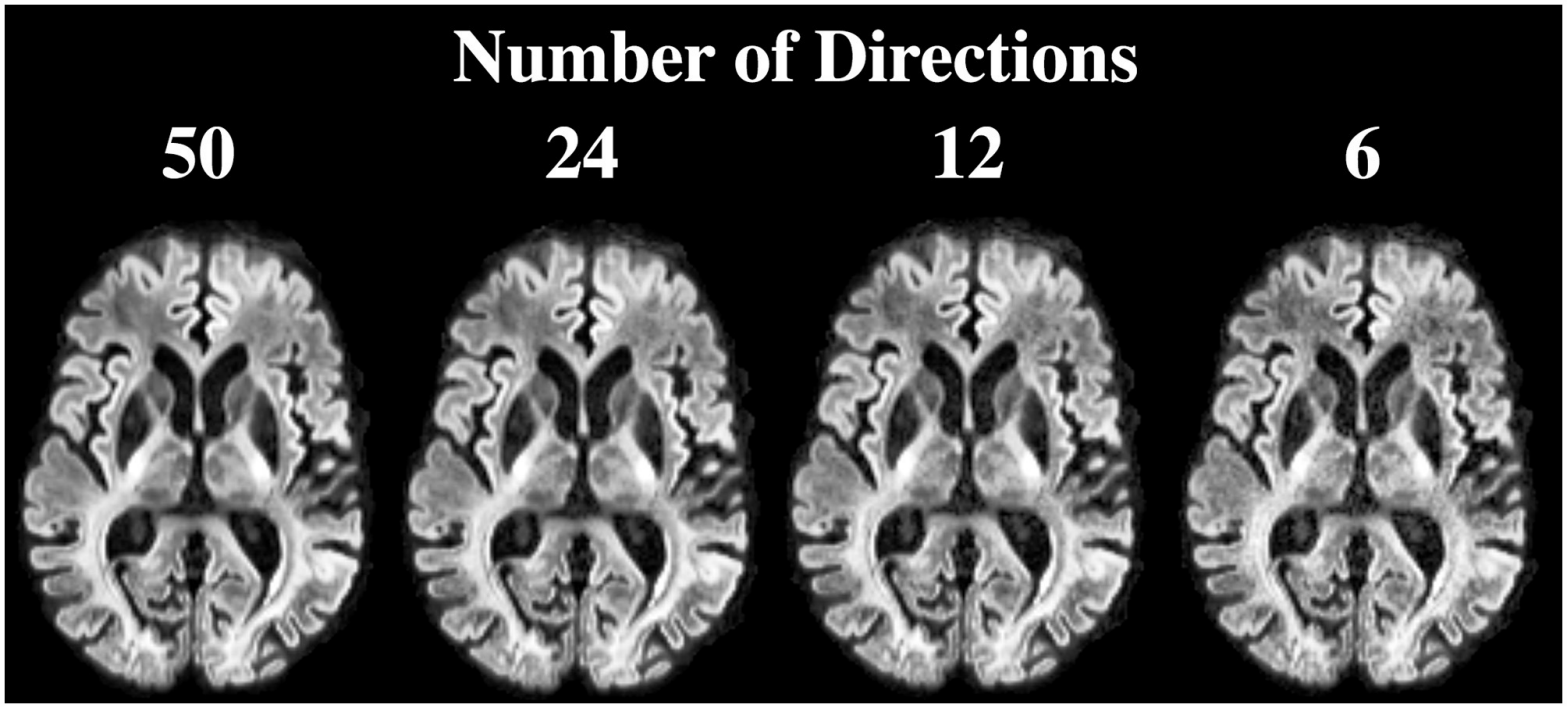
DiMANI contrast obtained using different numbers of directions and *b* = 1,500 s/mm^2^. Each dataset samples q-space uniformly.

Figure 8 shows the DiMANI contrast using different numbers of diffusion gradient directions from the same dataset. In our original data, we used 50 directions. However, the DiMANI contrast enables direct visualization of thalamic subnuclei even with a smaller number of directions. The main difference is the image noise level.

**Figure 9:**
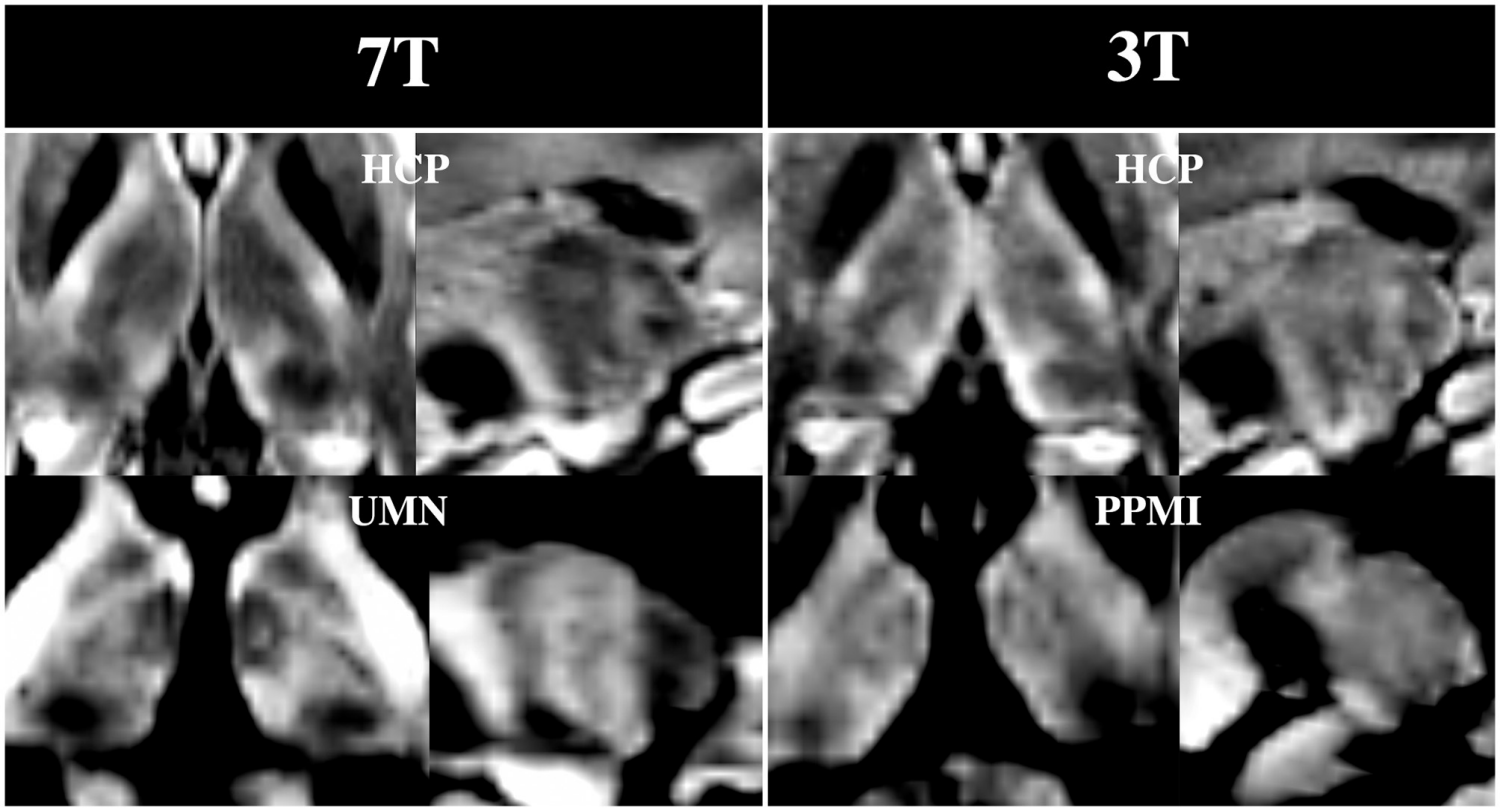
DiMANI images from multiple databases. Note that both HCP images were from dMRI sets acquired from the same control subject and shown for the *b* = 1,000 s/mm^2^ shell.

Figure 9 shows that many of the subnuclei are visible with the DiMANI contrast at two field strengths, different resolutions, and acquisition parameters. Both HCP datasets, coming from the same subject but at different field strengths, show repeatable features akin to those seen with the UMN data. The PPMI image, despite being acquired at 3T and being lower resolution, still enables the visualization of the zebra pattern in the sagittal view and several subnuclei in the axial.

## 4 Discussion

Here we present a new method for enhancing the anatomical contrast of subcortical tissue. The DiMANI method is based on averaging diffusion-weighted volumes, which allows one to directly visualize the subnuclei of the thalamus. Diffusion-based segmentation is becoming a field of interest as it enables faster acquisition of diffusion-related metrics for specific structures without relying on registration to a T1 or another modality. Few other studies have used a similar diffusion-based contrast to DiMANI; however, they focused on segmenting brain lesions (Liu et al., 2021), the gray matter ribbon (Little and Beaulieu, 2021), or the classic three-tissue type segmentation (white matter, gray matter, and cerebrospinal fluid) (Cheng et al., 2020). Although most current dMRI-based segmentation studies use state-of-the-art neural network technology, they are limited to the classical three-tissue based segmentation due to their use of common dMRI maps such as tensor and kurtosis results (Ciritsis et al., 2018; Cheng et al., 2020; Wang et al., 2020; Little and Beaulieu, 2021; Zhang et al., 2021). One other study created a deep learning algorithm to segment ten brain structures using dMRI data, but their ground truth was not based on segmentation from the dMRI data but rather warped atlas into diffusion space (Theaud et al., 2022). Due to the level of details exhibited, the DiMANI method has the potential to be used to generate gold standard ground truth for diffusion-based segmentation methods and improve current neural network approaches based on dMRI.

### 4.1 DiMANI contrast

In the DiMANI contrast, regions exhibiting dark signals are regions where water can diffuse isotropically (e.g., CSF) while bright regions reflect highly constrained water movement, or anisotropic diffusion (e.g., white matter tracts). The images from the present study show consistently and reliably different thalamic subnuclei with different levels of average diffusion signals reflecting the underlying histology of these subnuclei (Figures 2–4). Note that 50 directions randomly covering q-space (Caruyer et al., 2013) were averaged; therefore, it is unlikely that the contrast difference between any two subnuclei is due to any specific directions contained in the b-vectors. Further, we have shown that multiple datasets, using their own b-vector table, displayed similar DiMANI contrasts (Figure 9). The DiMANI contrast was also reliable (Figure 5A) and several raters were able to segment the subnuclei consistently (Figure 5B). Finally, the contrast was observed at multiple field strengths (Figure 9). It should be noted that different mathematical operations [mean, median, centromean (mean of middle 50% values), sum and the l2norm] yield similar contrasts with only few slight visual variations in in terms of noise and brightness; voxel values did differ across these different methods, but the visual contrast did not. In this study we chose the mean as this contrast is commonly used in DWI methods papers (Lee et al., 2021; Feizollah and Tardif, 2023).

### 4.2 Research potential

As shown in Figures 8, 9, the DiMANI contrast enables direct visualization of thalamic subnuclei with a variety of acquisition methods and parameters. This offers a clear advantage over methods that require the use of new sequences and the acquisition of new data. It enables researchers who have previously collected data to utilize this approach for thalamic parcellation. Further, a large number of ongoing neuroimaging studies already acquire dMRI data and no change of protocol would be required for these research teams to implement DiMANI in their pipeline. Additionally, using the DiMANI contrast to directly visualize and segment thalamic subnuclei facilitates the execution of diffusion-based research projects since no registration to other modalities or templates is necessary. Further, by eliminating registration errors and performing subject-specific segmentation, the accuracy of the representation of the anatomy is improved, thereby yielding more precise results when looking at quantitative diffusion metrics within specific subnuclei across groups.

### 4.3 Translational potential

We have shown in this work that thalamic subnuclei are directly visible in healthy controls as well as movement disorder patients using 7T and 3T, two field strengths currently FDA and CE approved for clinical work (Figure 9). Additionally, while having more diffusion directions clearly helps attain the appropriate signal-to-noise ratio (SNR), we have shown that usable results can be achieved with a smaller number of directions, which is more reasonable for a clinical setting (Figure 9). For thalamic-based therapies, such as DBS and focused ultrasound, direct visualization of the subnuclei could aid the targeting process by providing patient-specific information and enabling direct targeting (e.g., Vim for ET, anterior thalamus for epilepsy). Accurate direct targeting of thalamic subnuclei has the potential to reduce the heterogeneity in treatment outcome. Additionally, the ability to visualize the DBS electrodes with respect to the patient's actual anatomy could help clinicians decipher optimal stimulation settings more efficiently and provide context to the presence of side effects at lower thresholds in some patients. Further, the DiMANI contrast could potentially help identifying which thalamic subnuclei are affected when irregular thalamic anatomy such as atrophy or lesions are present. The method of averaging the diffusion weighted volumes is simple and is similar to “isotropic DWI” in the field of Radiology, a contrast that many scanners can generate. Therefore, there is no technical limitation for vendors to implement such contrast. The DiMANI method presented here is not proposed to replace existing methods, such as FGATIR. Instead, it is meant to be an additional tool available to directly visualize these elusive structures.

### 4.4 Limitations

One limitation worth noting is that DWI uses echo planar imaging (EPI-based) sequences which exhibit larger geometric distortions and susceptibility artifacts in the phase encoding direction. These are mainly problematic for surgical applications, such as pre-surgical planning for DBS and focused ultrasound. In our work, we acquired the diffusion data twice in a “blip up, blip down” configuration and processed the images using topup to mitigate this issue. The region of the thalamus, due to its central location, is less impacted by these distortions and as such the impact should be minimal compared to the cortex. One solution might be the use of multi-shot echoplanar diffusion MRI sequences that promise to result in sharper and largely distortion-free images, such as the FDA-approved syngo RESOLVE (https://www.siemens-healthineers.com/en-us/magnetic-resonance-imaging/options-and-upgrades/clinical-applications/syngo-resolve). Other solutions include gSlider BUDA-EPI and multi-coil dynamic B0 shimming (Liao et al., 2021) and MUSE (Chen et al., 2013). However, this does require longer acquisition times. While we have run this dMRI acquisition protocol on more than 100 DBS patients at 7T, we understand that acquiring about 13 min of dMRI data can be difficult in some patients. Future work will focus on optimizing DiMANI acquisition at 3T and 7T such that protocols could be seamlessly implemented in the clinic even in centers without a 7T scanner.

Manually segmenting a dozen or more subnuclei for every individual is extremely time consuming and potentially unfeasible. This is not problematic for clinical work since surgical teams only need to know the location of one or a few subnuclei depending on the application. For research, however, future work should focus on training deep learning algorithms specific to the DiMANI contrast to eliminate the need for manual segmentation. Finally, while the dMRI datasets presented here can be considered high resolution (1–1.25 mm isotropic), they are likely not able to resolve some of the smallest subnuclei found in histology and staining reports. Therefore, future studies should focus on acquiring higher resolution (sub-millimeter) dMRI data to uncover whether using DiMANI can enable direct visualization of more of the smaller subnuclei. Additionally, future work should focus on studying the impact of denoising tools on the DiMANI contrast and whether these tools can enable further optimization of data acquisition. Finally, follow-up studies should also evaluate whether the DiMANI contrast is able to depict variability in the size, shape and location of thalamic subnuclei among healthy subjects as well as those and in the diseased condition.

## 5 Conclusion

This study introduces the DiMANI contrast to directly visualize thalamic subnuclei using diffusion-weighted imaging. We have shown that this contrast is reliable and has a multitude of potential applications. DiMANI has great research potential as it can be applied to various acquisition methods and parameters, allowing researchers to utilize existing data to study the thalamus. Its direct visualization and segmentation capabilities eliminate the need for registration to other modalities, facilitating diffusion-based research projects. The translation to clinical applications of the DiMANI method may have significant impact as direct visualization of thalamic subnuclei can assist in the targeting and optimizations of thalamic-based neuromodulation therapies. In conclusion, this relatively simple method shows promising prospects and could be useful to the research and clinical community alike.

## Data availability statement

The raw data supporting the conclusions of this article will be made available by the authors, without undue reservation.

## Ethics statement

The studies involving humans were approved by University of Minnesota Internal Review Board. The studies were conducted in accordance with the local legislation and institutional requirements. The participants provided their written informed consent to participate in this study.

## Author contributions

RP: Conceptualization, Data curation, Formal analysis, Investigation, Methodology, Software, Visualization, Writing – original draft. TP: Data curation, Formal analysis, Investigation, Project administration, Software, Validation, Writing – review & editing. JC: Formal analysis, Investigation, Software, Validation, Writing – review & editing. KS: Investigation, Writing – review & editing. HB: Methodology, Software, Writing – review & editing. EY: Methodology, Writing – review & editing. RM: Investigation, Writing – review & editing. JA: Investigation, Writing – review & editing. SC: Investigation, Writing – review & editing. JV: Investigation, Writing – review & editing, Funding acquisition, Supervision. NH: Funding acquisition, Investigation, Methodology, Supervision, Validation, Writing – review & editing.
