# Supplementary material for: DiMANI: diffusion MRI for anatomical nuclei imaging—Application for the direct visualization of thalamic subnuclei
Source: Front Hum Neurosci. 2024 Feb 19;18:1324710. doi: 10.3389/fnhum.2024.1324710 (PMC10910100; doi:10.3389/fnhum.2024.1324710)
Supplement: Supplementary file 1 [file Table_1.DOCX]

*Supplementary Table1.* Thalamus groups, functions and therapy uses. VLP - ventral lateral posterior nucleus, VPL - Ventral posterior lateral nucleus, VLa - Ventral lateral anterior nucleus, VA - Ventral anterior nucleus, MD - Mediodorsal nucleus, MDmc – magnocellular division of MD, CM - Centromedian nucleus, LGN - Lateral geniculate nucleus, AV - Anterior ventral nucleus, MGN - Medial geniculate nucleus, AD - Anterior dorsal nucleus, AM - Anterior medial nucleus, CL - Central lateral nucleus, LD - Lateral dorsal nucleus, Li - Limitans nucleus, LP - Lateral posterior nucleus, Pf - Parafascicular nucleus, Pv - paraventricular nuclei, VM - Ventral medial nucleus. Note that several of these nuclei are further divided into more subnuclei (see Mai and Majtanik, 2018 and Boelens Keun et al., 2021 for more details).
